# SOD1 gains pro-oxidant activity upon aberrant oligomerization: change in enzymatic activity by intramolecular disulfide bond cleavage

**DOI:** 10.1038/s41598-022-15701-w

**Published:** 2022-07-11

**Authors:** Kosuke Yamazaki, Shinya Tahara, Takumi Ohyama, Kunisato Kuroi, Takakazu Nakabayashi

**Affiliations:** Graduate School of Pharmaceutical Sciences, Tohoku University, Sendai, Japan

**Keywords:** Molecular conformation, Bioinorganic chemistry, Biophysical chemistry

## Abstract

Copper-zinc superoxide dismutase (SOD1) has been proposed as one of the causative proteins of amyotrophic lateral sclerosis (ALS). The accumulation of non-native conformers, oligomers, and aggregates of SOD1 in motor neurons is considered responsible for this disease. However, it remains unclear which specific feature of these species induces the onset of ALS. In this study, we showed that disulfide-linked oligomers of denatured SOD1 exhibit pro-oxidant activity. Substituting all the cysteine residues in the free thiol state with serine resulted in the loss of both the propensity to oligomerize and the increase in pro-oxidant activity after denaturation. In contrast, these cysteine mutants oligomerized and acquired the pro-oxidant activity after denaturation in the presence of a reductant that cleaves the intramolecular disulfide bond. These results indicate that one of the toxicities of SOD1 oligomers is the pro-oxidant activity induced by scrambling of the disulfide bonds. Small oligomers such as dimers and trimers exhibit stronger pro-oxidant activity than large oligomers and aggregates, consistent with the trend of the cytotoxicity of oligomers and aggregates reported in previous studies. We propose that the cleavage of the intramolecular disulfide bond accompanied by the oligomerization reduces the substrate specificity of SOD1, leading to the non-native enzymatic activity.

## Introduction

**Figure 1:**
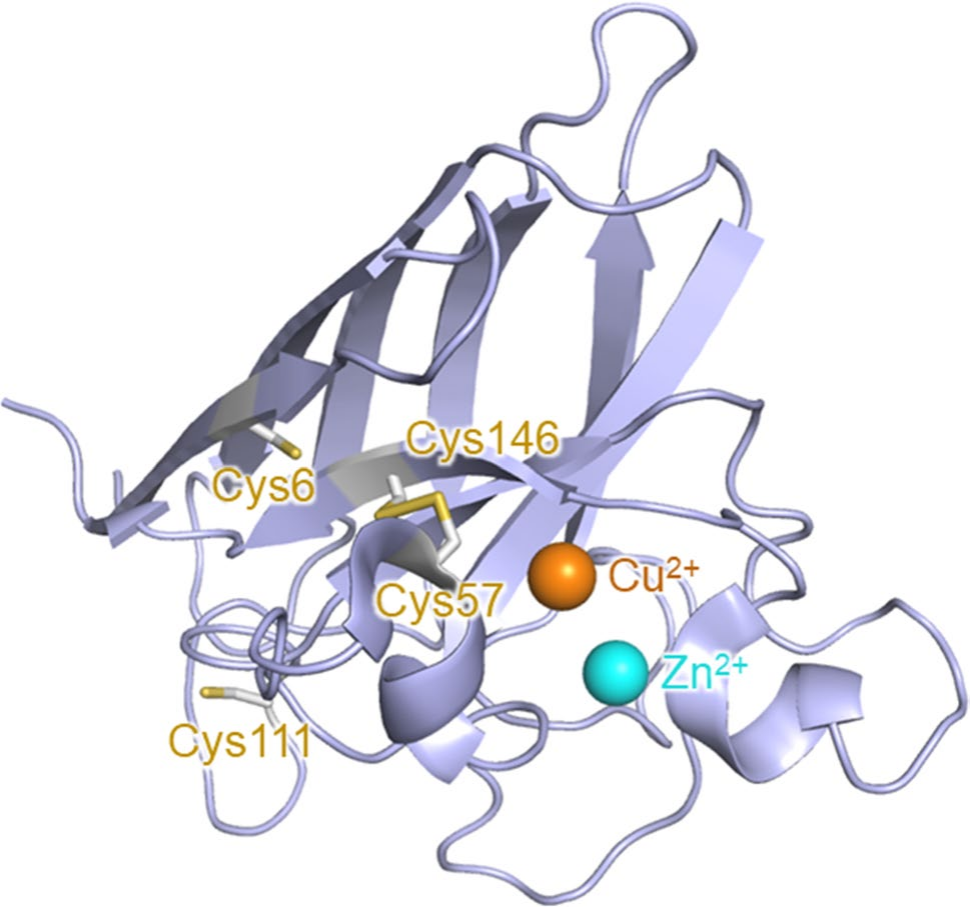
Crystallographic structure of the monomer unit of wild-type SOD1 (PDB code: 1PU0^26^). The Cu and Zn ions are shown as orange and light blue spheres, respectively. The cysteine residues are shown as sticks. Cys6 and Cys111 are in the free thiol state, while Cys57 and Cys146 form an intramolecular disulfide bond.

Amyotrophic lateral sclerosis (ALS) is a neurodegenerative disease that causes progressive muscle weakness and eventual death. The pathogenetic mechanism of ALS remains unclear, and currently there are no effective treatments^1,2^. Copper-zinc superoxide dismutase (SOD1), a metalloenzyme catalyzing the dismutation of superoxide radicals^3^, is first proposed to be involved in the onset of ALS because of the finding of SOD1 mutants in patients with familial ALS. Native human SOD1 is a homodimer, with each subunit containing one Cu ion and one Zn ion in the β-barrel structure (Fig. 1). The Cu-binding site acts as a catalytic site for the antioxidant activity, while the coordinated Zn ion contributes not to antioxidation but the stability of the protein structure^4^. Many studies have been conducted to understand the relationship between mutations of SOD1 and the pathogenesis of ALS, and it has been proposed that misfolded forms and aggregates of SOD1 mutants are the causative species of ALS^5–8^. SOD1 is very stable, and its denaturation temperature is above 75 °C; but removing Cu and Zn ions or reducing an intramolecular disulfide bond destabilizes the protein structure, resulting in denaturation even at physiological temperatures^4,9^. Mutations also reduce the stability of the protein and facilitate denaturation^4^, which is one of the possible reasons for the finding of SOD1 mutants in ALS patients. Several groups have proposed that wild-type (WT) SOD1 is also involved in the pathogenesis of sporadic ALS^10–12^. Indeed, misfolded WT was detected in ALS patients and model mice^8,13,14^.

SOD1 has been proposed to exhibit toxic properties upon forming large aggregates such as fibrils and small oligomers such as trimers^5,6,15,16^. Insoluble large aggregates and fibrils were previously considered to be important in the development of ALS^5,6^, as in other neurodegenerative diseases such as Alzheimer’s and Parkinson’s diseases^17^. However, it was recently reported that ALS-related mutants do not exhibit an apparent correlation between their aggregation propensity and the disease duration of ALS patients^18^. Furthermore, recent studies have shown that trimer-stabilizing mutants of SOD1 strongly promote cell death^15,16^ compared to aggregation-prone mutants. These results suggest that the SOD1 oligomers are the origin of the cytotoxicity and that the aggregate formation may rather play a protective role in cells^16^. However, the molecular mechanism by which the non-native oligomers exert their toxicity remains unclear.

Several groups have studied the architectures of the non-native oligomers of SOD1^19–23^. Previous studies showed that short segments of SOD1 are prone to assemble into oligomers and fibrils by the formation of intramolecular hydrogen bonds and hydrophobic interactions^21,22^. SOD1 also forms non-native oligomers in which monomers are linked via intermolecular disulfide bonds^19,20^. Breaking the intramolecular disulfide bond in SOD1 is important for the oligomerization^20^. SOD1 possesses four cysteine residues, and Cys6 and Cys111 exist as the free thiol state while Cys57 and Cys146 form an intramolecular disulfide bond in the native conformation (Fig. 1)^24^. Denaturation allows Cys6 and Cys111 to attack the Cys57–Cys146 disulfide bond, promoting the formation of the intermolecular disulfide bonds involving Cys57 and Cys146^20^.

It has been shown that the ALS-related mutants of SOD1 exhibit oxidative properties^25–29^. We have studied the toxicity of SOD1 in terms of its oxidation properties and shown that the denatured SOD1 exhibits strong pro-oxidant activity, which generates reactive oxygen species (ROS) including hydroxyl radicals from hydrogen peroxide^30–32^. The ALS-related SOD1 mutants from which Cu and Zn ions were removed (apo-SOD1) were readily denatured at 37 °C, and these denatured species exhibit the strong pro-oxidant activity after rebinding Cu ions. The rebinding of Cu ions promoted local refolding of the denatured SOD1, forming a catalytic center that is largely different from the native one^31,33,34^. Such oxidative behavior was also observed for apo-WT. Apo-WT was not denatured at 37 °C; however, a macromolecular crowding environment mimicking intracellular conditions destabilized the protein structure, resulting in the denaturation and acquisition of the pro-oxidant activity at physiological temperatures^35^. In the present study, we show that SOD1 acquires the pro-oxidant activity upon forming the non-native disulfide-linked oligomers. Small oligomers such as dimers and trimers exhibited stronger pro-oxidant activity than large oligomers and aggregates. These results indicate that the pro-oxidant activity gives rise to the toxicity of the small SOD1 oligomers. Furthermore, we propose the molecular mechanism that the cleavage of the intramolecular disulfide bond associated with the oligomerization leads to the acquisition of the pro-oxidant activity.

## Experimental methods

### Sample preparation

The preparations of recombinant human SOD1 and its mutants were previously described^36^. Briefly, SOD1 with an N-terminal hexahistidine tag and a thrombin cleavage site was overexpressed in *Escherichia coli* BL21(DE3) strain (Agilent). The proteins were purified by Ni–NTA affinity chromatography (Ni Sepharose 6 Fast Flow, Cytiva). Zn ions were added to the obtained solutions, and the proteins were further purified by salting out. The purified protein solutions were treated with thrombin (Cytiva) to remove the hexahistidine tag, and then SOD1 without the hexahistidine tag was isolated by Ni–NTA chromatography (HisTrap HP, Cytiva). The protein solutions were finally dialyzed against acetate buffer containing ethylenediaminetetraacetic acid (EDTA) to obtain the apo form of SOD1 and its mutants. The sample concentration was adjusted to 20 μM in monomer units unless stated otherwise.

### Denaturation and oligomerization of SOD1

The apo forms of SOD1 and its mutants were denatured by incubating in 50 mM phosphate buffer at pH 7.5. The apo-WT solution was incubated for 24 h at 45 °C, and the apo-A4V, apo-H43R, apo-G93A, and apo-G147P solutions were incubated for 90 min at 37 °C. The apo-A4V/C6S/C111S, apo-H43R/C6S/C111S, and apo-G93A/C6S/C111S solutions were incubated for 24 h at 45 °C with and without 10 mM dithiothreitol (DTT). After the incubation, DTT was removed using a desalting column (PD-10, Cytiva). Cleavage of the intramolecular disulfide bond after the DTT treatment was confirmed by the 5,5′-dithio-bis-(2-nitrobenzoic acid) (DTNB) assay (described below). CD spectra before and after the incubations were recorded using a spectropolarimeter (J-820, JASCO). Non-reducing SDS-PAGE analyses were performed using 15% polyacrylamide gels. These experiments were carried out 3 times for all the SOD1 samples.

### 5,5′-Dithio-bis-(2-nitrobenzoic acid) (DTNB) assay

Solutions of the SOD1 mutants were mixed with an ethanol solution of DTNB (Ellman’s reagent). The final concentration of DTNB was 100 μM. The solutions were incubated for 30 min at room temperature, and then their absorption spectra were recorded. The absorbance at 412 nm due to 5-mercapto-2-nitrobenzoic acid (TNB, ε_412_ = 15,500) was used to evaluate the number of thiol groups. These experiments were carried out 3 times for all the SOD1 samples, and standard errors (SE, *n* = 3) were calculated.

### Evaluation of pro-oxidant activity

Fluorescence assays using dichlorofluorescein (DCF) were carried out to evaluate the pro-oxidant activity of SOD1^30^. 2′,7′-Dichlorofluorescein diacetate (DCFH-DA) was purchased (Wako) and used as received. DCFH was prepared by hydrolysis of DCFH-DA in a NaOH solution. DCFH was then mixed with H_2_O_2_. A 3-fold molar excess of CuCl_2_ over the SOD1 monomer was added to the apo-SOD1 solution and mixed. The final concentrations of DCFH, H_2_O_2_, CuCl_2_, and SOD1 were 50 μM, 50 μM, 30 μM, and 10 μM, respectively. The obtained mixed solutions were incubated at room temperature for 5 min. Fluorescence spectra of these solutions were recorded using a fluorescence spectrophotometer (FP-6500, JASCO), and the peak intensity was adopted as the quantitative measure of the pro-oxidant activity. The excitation wavelength was 495 nm. These experiments were carried out 3 times for all the SOD1 samples, and SE (*n* = 3) were calculated.

### Size exclusion chromatography

Size exclusion chromatography (Superose 12 10/300 GL and ӒKTA prime plus, Cytiva) was carried out to separate oligomers of different molecular weights. An apo-G93A solution was incubated for 90 min at 37 °C to induce oligomerization. The concentrations of the obtained oligomer solutions were adjusted to 20 μM in monomer units, and DCF fluorescence measurements were carried out to evaluate their pro-oxidant activity. These experiments were carried out 3 times for all the SOD1 samples.

### Cleavage of the Cys57–Cys146 disulfide bond and its effect on pro-oxidant activity

A DTT solution was added to the solution of the G93A mutant to a final concentration of 10 mM. Subsequently, the solution was incubated for 24 h at 4 °C. Then DTT was removed using a desalting column (PD-10, Cytiva). Cleavage of the intramolecular disulfide bond after the DTT treatment was confirmed by the DTNB assay. The concentration of the obtained solution was adjusted to 20 μM in monomer units, and the pro-oxidant activity was evaluated by DCF fluorescence measurements. These experiments were carried out 3 times for all the SOD1 samples, and SE (*n* = 3) were calculated.

## Results

We examined the denaturation, oligomerization, and pro-oxidant activity of WT and the A4V, H43R, G93A, and G147P mutants. The three mutants, A4V, H43R, and G93A are regarded as the ALS-related mutants causing the rapid progression of ALS. The G147P mutant was previously reported to readily form small oligomers such as trimers and gradually form aggregates under physiological conditions^15^. We also prepared the A4V/C6S/C111S, H43R/C6S/C111S, and G93A/C6S/C111S mutants, in which all the cysteine residues in the free thiol state are replaced with serine (Fig. 1). These cysteine mutants are expected to be deficient in the ability of oligomerization. We also examined the denaturation of these cysteine mutants in the presence of 10 mM dithiothreitol (DTT). This treatment is expected to restore the oligomerization propensity of these mutants because the Cys57–Cys146 disulfide bond is reduced, generating free thiol groups that can form intermolecular disulfide bonds. The 5,5′-dithio-bis-(2-nitrobenzoic acid) (DTNB) assay confirmed the generation of free thiol groups in SOD1 by the DTT treatment (Fig. S1).

**Figure 2:**
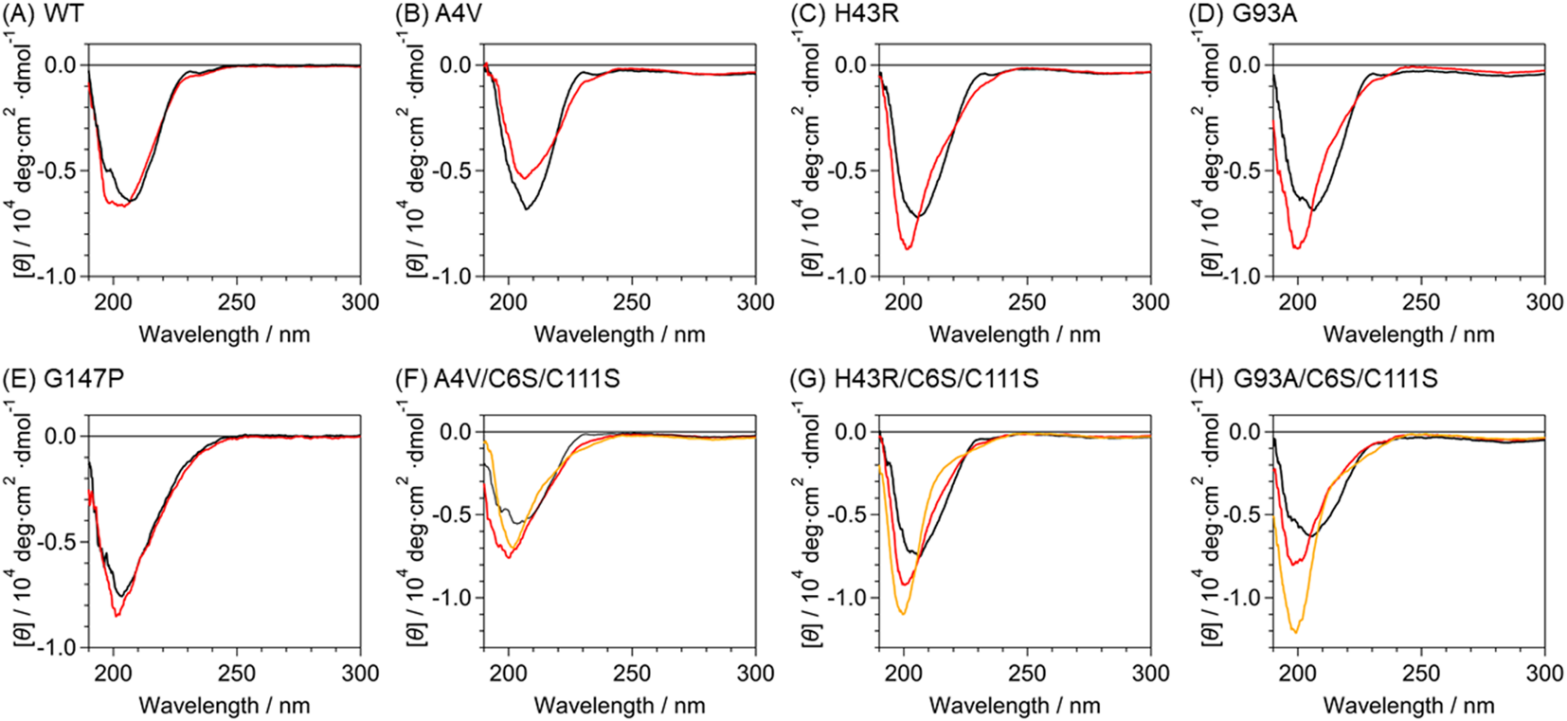
CD spectra of (**A**) apo-WT, (**B**) apo-A4V, (**C**) apo-H43R, (**D**) apo-G93A, (**E**) apo-G147P, (**F**) apo-A4V/C6S/C111S, (**G**) apo-H43R/C6S/C111S, and (**H**) apo-G93A/C6S/C111S in 50 mM phosphate buffer at pH 7.5 before and after the incubation (black and red lines, respectively). Details of the incubation conditions are given in Materials and methods and in the text. Panels F–H also show the CD spectra of the mutants after the incubation with 10 mM DTT (orange lines). DTT was removed using a desalting column before the CD measurement.

SOD1 acquires aggregation propensity and pro-oxidant activity upon denaturation. We previously reported that the mutants A4V, H43R, and G93A were denatured in the apo-form in phosphate buffer upon incubation for 90 min at 37 °C^30–34^, and the denaturation of apo-WT required incubation for 24 h at 45 °C^35^. Circular dichroism (CD) spectroscopy enables us to confirm protein denaturation (Fig. 2). The CD spectrum of WT (Fig. 2A) after the incubation for 24 h at 45 °C showed a shift of the negative band from 210 to 200 nm accompanied by an increase in its amplitude. This spectral change reflects the increase in the content of a random coil structure due to the denaturation. Apo-H43R and apo-G93A also exhibited the same behavior assignable to the denaturation after the incubation for 90 min at 37 °C (Fig. 2C,D). These results are the same as those previously reported^31^. Only apo-A4V showed a decrease in the amplitude of the band at 205 nm (Fig. 2B). This spectral change was also observed in a previous study and attributed to the efficient aggregation of apo-A4V^31^.

The CD spectrum of apo-G147P exhibited a negative band at around 200 nm already before the incubation (Fig. 2E). This result indicates that apo-G147P formed a denatured structure even before the incubation. Since Gly147 is located adjacent to Cys146, the G147P mutation likely prevents the formation of the Cys57–Cys146 intramolecular disulfide bond and facilitates protein misfolding. The slight increase in the band intensity at around 200 nm indicates that the incubation further promoted the denaturation of G147P.

The apo-A4V/C6S/C111S, apo-H43R/C6S/C111S, and apo-G93A/C6S/C111S mutants did not show denaturation after the incubation for 90 min at 37 °C (Fig. S2). This result implies the increase in the structural stability by removing the cysteine residues in the free thiol state. After the incubation for 24 h at 45 °C, these mutants showed an increase in the amplitude of the CD spectrum at 200 nm, confirming the denaturation of these mutants (Fig. 2F–H). The spectral changes of the cysteine mutants due to the denaturation are similar to those of apo-WT and the ALS-related mutants. We also confirmed the denaturation of these mutants after the incubation for 24 h at 45 °C in the presence of 10 mM DTT (Fig. 2F–H).

**Figure 3:**
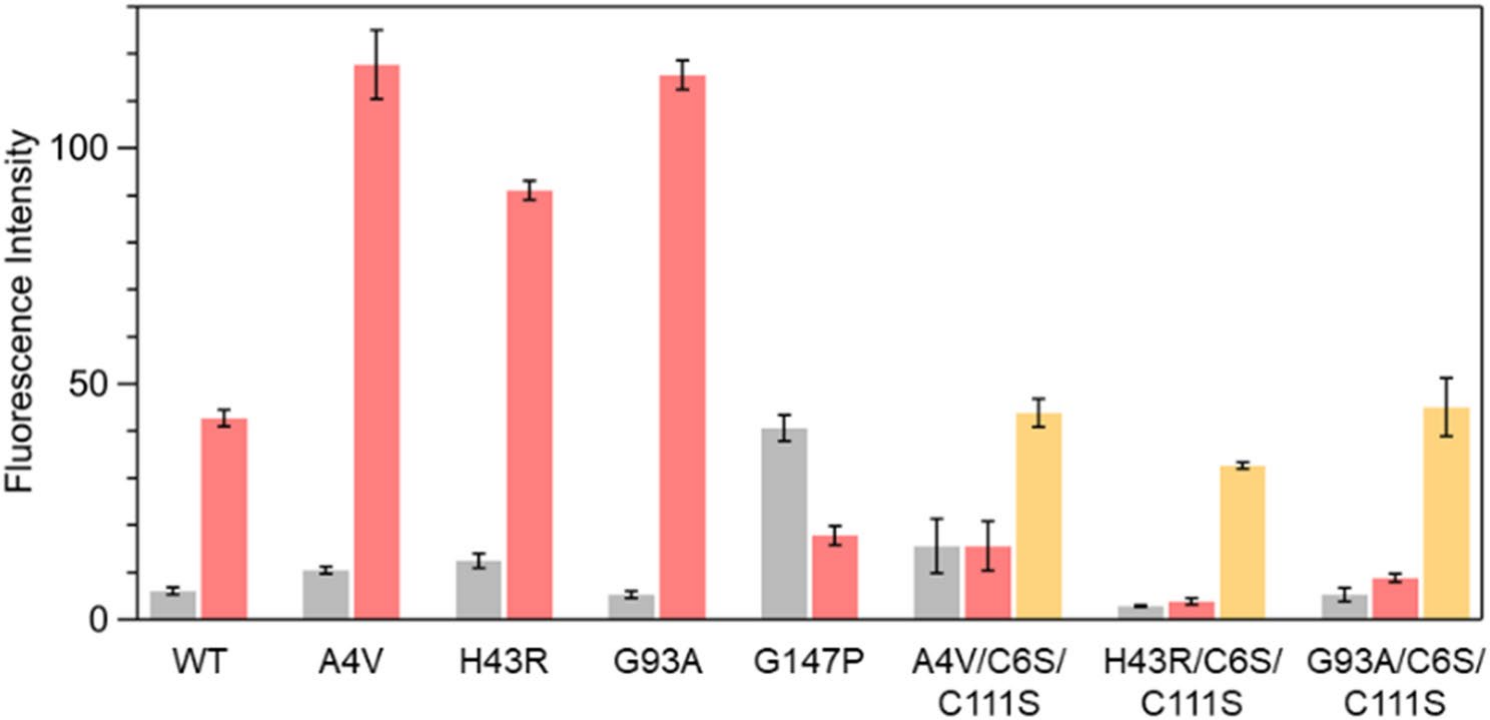
DCF fluorescence intensities of apo-WT, apo-A4V, apo-H43R, apo-G93A, apo-G147P, apo-A4V/C6S/C111S, apo-H43R/C6S/C111S, and apo-G93A/C6S/C111S. Gray and red bars show the fluorescence intensities before and after the incubation, respectively. Orange bars show those after the incubation with 10 mM DTT. Detailed information on the incubation conditions is given in the Materials and Methods Section and in the text. Error bars are SE (*n* = 3).

We then carried out the fluorescence assay^31^ to evaluate the magnitude of the pro-oxidant activity of SOD1 before and after the denaturation (Fig. 3). We previously showed that apo-A4V, apo-H43R, and apo-G93A acquire the pro-oxidant activity after the denaturation and the subsequent binding of Cu ions^31^. We thus added a 3-fold molar excess of Cu ions relative to the SOD1 monomer so that Cu ions entirely occupied all the metal-binding sites. Complete binding of Cu ions to apo-A4V, apo-H43R, and apo-G93A was confirmed in previous studies^31,33^, and it was confirmed for apo-A4V/C6S/C111S, apo-H43R/C6S/C111S, and apo-G93A/C6S/C111S by measurements of CD spectra (Fig. S3). Then H_2_O_2_ and dichlorodihydrofluorescein (DCFH) were added to the SOD1 solution. If SOD1 possesses pro-oxidant activity, ROS are generated through the catalytic reaction of SOD1, resulting in the oxidation of DCFH to strong fluorescent dichlorofluorescein (DCF). We used the peak intensity of the fluorescence spectrum of DCF as a quantitative measure of pro-oxidant activity.

The DCF fluorescence intensity of apo-WT, apo-A4V, apo-H43R, and apo-G93A increased by 7-, 11-, 8-, and 23-fold, respectively, after the incubation and the subsequent Cu ion binding, indicating that these proteins acquired the pro-oxidant activity after the denaturation. Apo-A4V/C6S/C111S, apo-H43R/C6S/C111S, and apo-G93A/C6S/C111S showed no substantial increase in the DCF fluorescence intensity after the incubation. This result indicates that these cysteine mutants do not acquire pro-oxidant activity after the denaturation. The absence of the pro-oxidant activity even after the denaturation indicates that the pro-oxidant activity is not solely due to the denaturation and that other factors are involved in the change in the enzymatic activity to the pro-oxidation. In contrast, the DCF fluorescence intensity of these cysteine mutants increased by 3, 11, and 8 times after the denaturation in the presence of DTT, suggesting a relationship between the state of the intramolecular disulfide bond and the pro-oxidant activity. Apo-G147P was insensitive to the addition of Cu ions in its CD spectrum, which prevented us from confirming the binding of Cu ions to apo-G147P; however, the addition of Cu ions induced the marked pro-oxidant activity in G147P before the incubation, which is consistent with the denatured structure before the incubation (Fig. 2).

**Figure 4:**
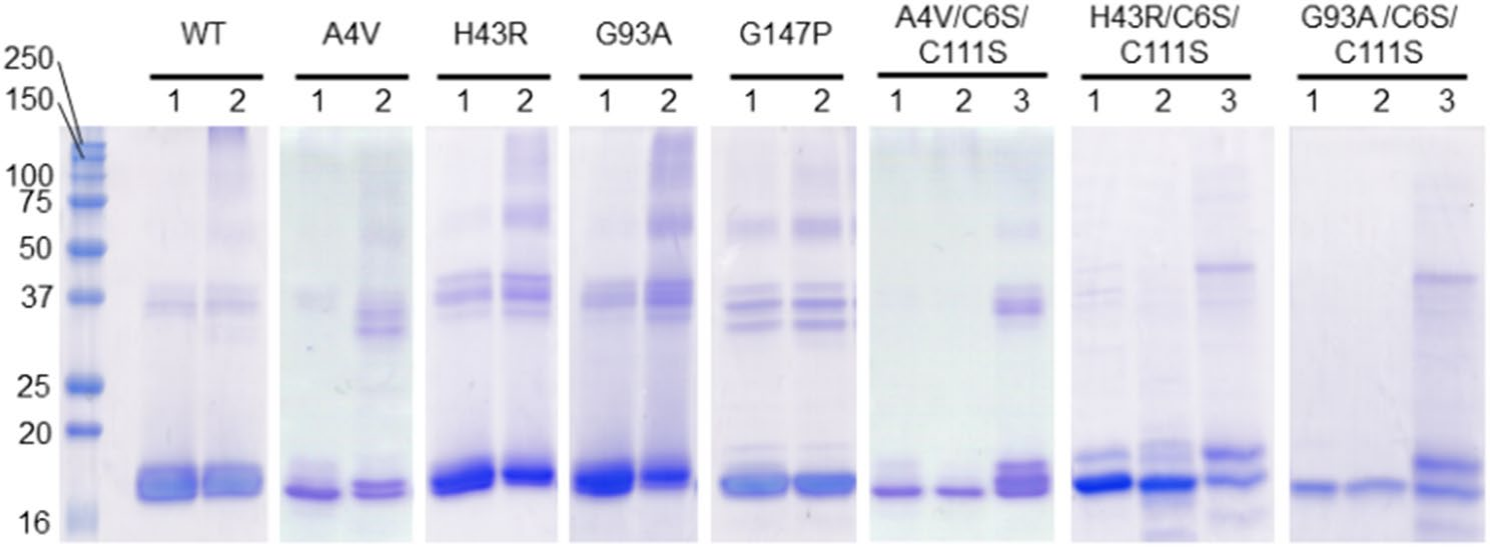
Non-reducing SDS-PAGE of apo-WT, apo-A4V, apo-H43R, apo-G93A, apo-G147P, apo-A4V/C6S/C111S, apo-H43R/C6S/C111S, and apo-G93A/C6S/C111S in 50 mM phosphate buffer at pH 7.5. Lanes: 1, before incubation; 2, after incubation; 3, after incubation with 10 mM DTT. Details of the incubation conditions are given in Materials and methods and in the text.

We carried out SDS-PAGE to examine the oligomerization state of SOD1 before and after the incubation (Fig. 4, full-length SDS-PAGE results with molecular weight markers are shown in Fig. S4). All the proteins showed a band at a molecular weight slightly higher than 16 kDa, which is assignable to the SOD1 monomer. Upon boiling and treatment with SDS in the analysis, the native SOD1 dimer dissociates into two monomers. After the incubation, WT, apo-A4V, apo-H43R, and apo-G93A exhibited multiple bands at around and above 37 kDa, indicating the formation of oligomers with various apparent molecular weights. As in previous studies, the bands at around 37 kDa and slightly above 50 kDa were assigned to the dimers and trimers, respectively^19^. Large oligomers were also observed at > 75 kDa. These results indicate that the denaturation of SOD1 promotes the formation of the non-native oligomers. Comparisons of SDS-PAGE results between with and without reducing treatments (Fig. S5) showed that oligomeric bands were not clearly seen in SDS-PAGE after reducing the samples, indicating that the oligomers are generated by formations of intermolecular disulfide bonds. Observing the multiple and broad bands implies the structural heterogeneity of these oligomers, which likely arise from intermolecular disulfide bonds between different cysteine residues. We note that, even before the incubation, faint bands were observed at around 37 kDa. These bands might be due to non-native dimers formed during the sample treatment for SDS-PAGE. Apo-G147P showed bands due to oligomers both before and after the incubation. Apo-G147P was denatured before the incubation, consistent with the understanding that the denaturation promotes oligomerization of SOD1.

The monomer bands were dominantly observed for apo-A4V/C6S/C111S, apo-H43R/C6S/C111S, and apo-G93A/C6S/C111S both before and after the incubation. This result is consistent with the expectation that oligomers cannot be formed by proteins without free cysteine residues because they cannot form intermolecular disulfide bonds. On the other hand, these mutants are oligomerized after the incubation with DTT (lane 3 in Fig. 4). The generation of the thiol groups upon reduction of the intramolecular disulfide bond enabled the formation of the intermolecular disulfide bonds. These results indicate that the free thiol groups are necessary for the formation of non-native oligomers.

The present results show a strong correlation between the oligomerization propensity and the pro-oxidant activity. Apo-WT, apo-A4V, apo-H43R, and apo-G93A efficiently formed the oligomers and acquired the strong pro-oxidant activity upon the denaturation, while neither oligomerization nor acquisition of the pro-oxidant activity was observed for the mutants with C6S/C111S mutation. However, after denaturation in the presence of the reductant, these cysteine mutants formed the oligomers and acquired the pro-oxidant activity. These results provide evidence that SOD1 acquires the pro-oxidant activity upon oligomerization. In other words, the oligomers of SOD1 possess the pro-oxidant activity that can increase oxidative stress in cells.

We further investigated the relationship between the oligomer size and the pro-oxidant activity. Apo-H43R and apo-G93A were incubated, and the oligomers with different molecular weights were separated by size exclusion chromatography. We could not isolate each oligomer but obtained eluted fractions with different oligomer compositions (Fig. S6). The concentrations of the obtained fractions were adjusted to 20 μM in monomer units, and DCF fluorescence measurements were performed. Fractions that dominantly contained dimers and trimers exhibited stronger pro-oxidant activity than those mainly containing monomers and large oligomers (Fig. S6). Our results indicate that the strong toxicity of the SOD1 trimers^15,16^ is rationally explained by oxidative stress.

## Discussion

**Figure 5:**
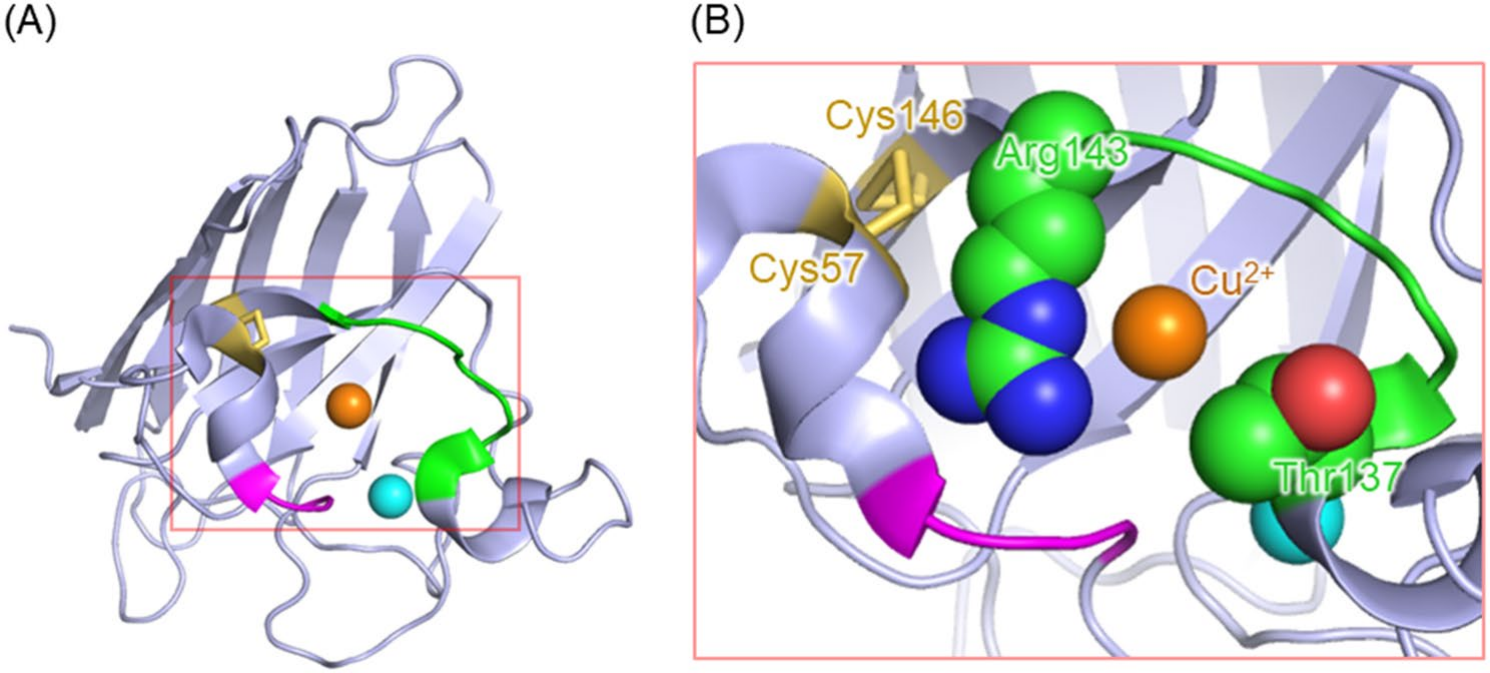
Overall structure and catalytic site of wild-type SOD1 (PDB code: 1PU0^26^). (**A**) Overall structure. (**B**) Close-up of the region indicated with a red box in (**A**) which includes the Cu-binding site (orange sphere), the intramolecular disulfide bond (yellow sticks), and the Zn^-^binding and electrostatic loops (pink and green loops).

**Figure 6:**
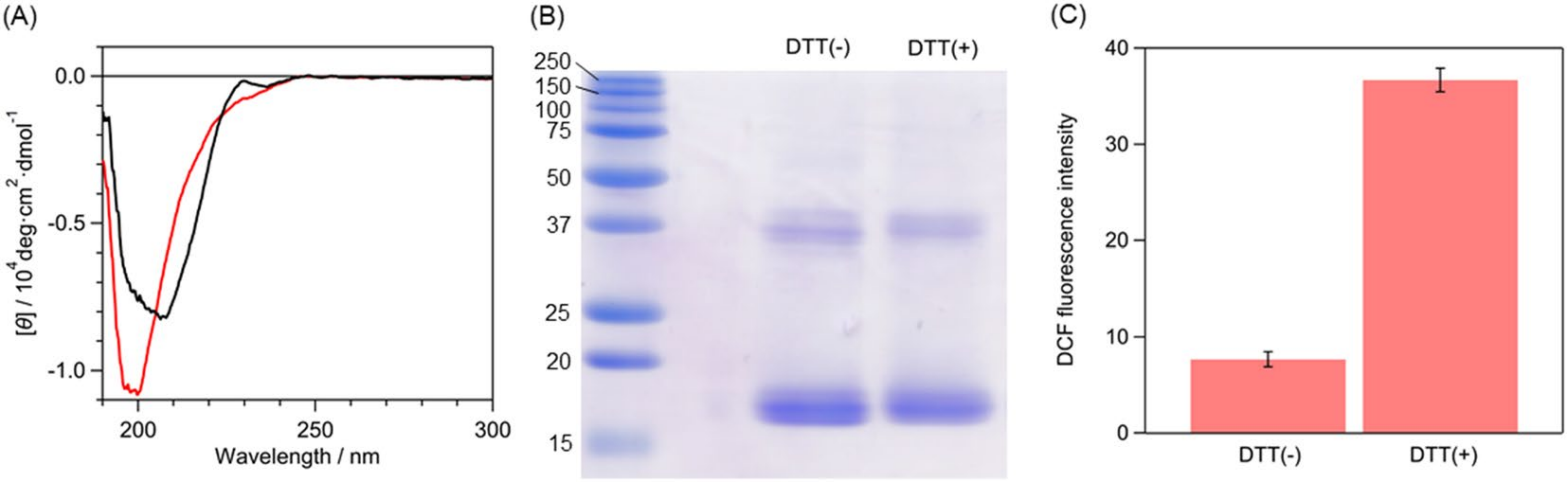
Reduction of the intramolecular disulfide bond of apo-G93A. (**A**) CD spectra, (**B**) non-reducing SDS-PAGE, and (**C**) DCF fluorescence intensities of apo-G93A after the incubation at 4 °C for 24 h in the presence and absence of 10 mM DTT. In Panel (**A**), CD spectra before and after the DTT treatment are shown as red and black lines. In Panel (**C**), error bars are SE (*n* = 3).

In the present study, we showed that SOD1 acquires the pro-oxidant activity upon the oligomerization. The pro-oxidant activity was weaker for large oligomers and aggregates and was not detected for the cysteine mutants unable to oligomerize. However, these cysteine mutants acquired the pro-oxidant activity after cleaving the intramolecular disulfide bonds and subsequent oligomer formation. These results indicate that one of the toxicities specific to SOD1 oligomers is the pro-oxidant activity. We discuss the molecular mechanism underlying the pro-oxidant activity of SOD1 using the crystallographic structure of the monomer unit of WT (Fig. 5)^24^. A Cu ion is bound in the cavity composed of a β-barrel structure and two loops called the Zn-binding and electrostatic loops. X-ray crystallographic and mutagenesis studies showed that Thr137 and Arg143 in the electrostatic loop are the important residues for the substrate specificity of SOD1 that enables selective dismutation of O_2_^−^^37,38^. Thr137 limits the size of the substrate by steric effects^38^, and Arg143 selectively attracts anions by electrostatic interactions^37^. The Cys57–Cys146 intramolecular disulfide bond links the Zn-binding and electrostatic loops, and the oligomerization process involves cleavage of the Cys57–Cys146 bond^20^. It was shown that the electrostatic loop is disordered in the absence of the Cys57–Cys146 bond by small-angle X-ray scattering and H/D exchange reactions detected by NMR^39^. Our group also reported that, using UV resonance Raman spectroscopy, the acquisition of the pro-oxidant activity accompanies the loss of the dative bond between the Cu ion and His120^31,34^, which is hydrogen-bonded with Gly141 and Ser142 in the electrostatic loops^40^. This result suggests that significant structural rearrangements take place around the catalytic site upon the oligomerization. The disorder of the electrostatic loop may cause the loss of the original substrate specificity, allowing H_2_O_2_ to approach the catalytic center and to be oxidized. To test this hypothesis, we reduced the Cys57–Cys146 bond at 4 °C of apo-G93A (Fig. S7) and investigated its pro-oxidant activity. The cleavage of the Cys57–Cys146 bond led to destabilization of the protein structure (Fig. 6A), but did not induce oligomer formation (Fig. 6B). Apo-G93A after the Cys57–Cys146 cleavage exhibited a 5-fold increase in the DCF fluorescence intensity (Fig. 6C), meaning the acquisition of the pro-oxidant activity. This result indicates that breaking the Cys57–Cys146 bond is an essential molecular step for the acquisition of the pro-oxidant activity of SOD1. Small oligomers such as dimers or trimers exhibited stronger pro-oxidant activity than large oligomers or aggregates. In large oligomers and aggregates, most of the catalytic sites of the monomeric units of SOD1 are likely buried, giving rise to the decreased accessibility of H_2_O_2_ to the catalytic site.

The present study showed that SOD1 requires denaturation, oligomerization, and subsequent Cu ion binding to acquire the pro-oxidant activity. The pro-oxidant activity of SOD1 oligomers likely induces cellular oxidative stress; typical oxidative chemical species are known to accumulate intracellularly in the pathogenesis of ALS^41^. In fact, ROS generation^42^, accumulation of oxidized species of proteins, DNA, membrane phospholipids, and thiol compounds have been shown in several model systems^42–45^, and impairment of the redox homeostasis was also observed^45–47^. Thus the next step in this study will be to investigate whether SOD1 oligomers induce ALS-like redox aberration in cells. It was previously reported that the homeostasis of Zn ions is impaired in the spinal cords of ALS patients due to the low expression level of Zn transporters^48^. Furthermore, the Zn-binding affinity of most ALS-related mutants is reduced compared with that of WT^49,50^. It is thus plausible that a fraction of SOD1 proteins does not bind Zn ions and exists in the Zn-deficient form in ALS patients. Zn-deficient SOD1 is thermally unstable and readily undergoes denaturation^4^. Therefore, it would be very important to investigate the relationship between intracellular Zn ion concentrations and SOD1 cytotoxicity in relation to oligomer formation.

## Conclusion

We investigated the relationship between the formation of the disulfide-linked oligomers and the oxidative property of SOD1. Wild-type SOD1 and its mutants exhibited the oligomerization and the pro-oxidant activity after denaturation. Mutations of cysteine residues possessing the free thiol group impaired the oligomerization and the increase in the pro-oxidant activity after denaturation. In contrast, these cysteine mutants oligomerized and exhibited the pro-oxidant activity after denaturation in the presence of a reductant that cleaves the intramolecular disulfide bond. These results clearly indicate that the disulfide-linked oligomers of SOD1 exhibit the pro-oxidant activity. We also found that small SOD1 oligomers such as dimers and trimers exhibited stronger pro-oxidant activity than large oligomers and aggregates. Previous studies showed that trimer-stabilizing SOD1 mutants strongly promote cell death compared to aggregation-prone mutants^15,16^. The present study indicates that one of the toxicities of SOD1 trimers is the pro-oxidant activity. Furthermore, we proposed the molecular mechanism by which SOD1 gains the pro-oxidant activity upon formation of the disulfide-linked oligomers. The formation of the disulfide-linked oligomers involves the cleavage of the intramolecular disulfide bond, which leads to the destabilization of the Zn-binding and electrostatic loops^39^. The disorder of these two loops reduces the substrate specificity and changes the enzymatic activity of SOD1.

## Supplementary Information

Supplementary Information.

## Data Availability

All data acquired and analyzed in this study are included in this paper and the Supplementary Information file.

## References

[1] Taylor JP, Brown RH, Cleveland DW (2016). Decoding ALS: From genes to mechanism. Nature.

[2] Jaiswal MK (2019). Riluzole and edaravone: A tale of two amyotrophic lateral sclerosis drugs. Med. Res. Rev..

[3] Fukai T, Ushio-Fukai M (2011). Superoxide dismutases: Role in redox signaling, vascular function, and diseases. Antioxid. Redox Signal..

[4] Furukawa Y, O'Halloran TV (2005). Amyotrophic lateral sclerosis mutations have the greatest destabilizing effect on the apo- and reduced form of SOD1, leading to unfolding and oxidative aggregation. J. Biol. Chem..

[5] Wang J, Slunt H, Gonzales V, Fromholt D, Coonfield M, Copeland NG, Jenkins NA, Borchelt DR (2003). Copper-binding-site-null SOD1 causes ALS in transgenic mice: Aggregates of non-native SOD1 delineate a common feature. Hum. Mol. Genet..

[6] Wang Q, Johnson JL, Agar NYR, Agar JN (2008). Protein aggregation and protein instability govern familial amyotrophic lateral sclerosis patient survival. Plos Biol..

[7] Paré B, Lehmann M, Beaudin M, Nordström U, Saikali S, Julien J-P, Gilthorpe JD, Marklund SL, Cashman NR, Andersen PM, Forsberg K, Dupré N, Gould P, Brännström T, Gros-Louis F (2018). Misfolded SOD1 pathology in sporadic Amyotrophic Lateral Sclerosis. Sci. Rep..

[8] Tokuda E, Takei Y-I, Ohara S, Fujiwara N, Hozumi I, Furukawa Y (2019). Wild-type Cu/Zn-superoxide dismutase is misfolded in cerebrospinal fluid of sporadic amyotrophic lateral sclerosis. Mol. Neurodegener..

[9] Sekhar A, Rumfeldt JAO, Broom HR, Doyle CM, Bouvignies G, Meiering EM, Kay LE (2015). Thermal fluctuations of immature SOD1 lead to separate folding and misfolding pathways. Elife.

[10] Bosco DA, Morfini G, Karabacak NM, Song Y, Gros-Louis F, Pasinelli P, Goolsby H, Fontaine BA, Lemay N, McKenna-Yasek D, Frosch MP, Agar JN, Julien J-P, Brady ST, Brown RH (2010). Wild-type and mutant SOD1 share an aberrant conformation and a common pathogenic pathway in ALS. Nat. Neurosci..

[11] Rotunno MS, Bosco DA (2013). An emerging role for misfolded wild-type SOD1 in sporadic ALS pathogenesis. Front. Cell. Neurosci..

[12] Grad LI, Yerbury JJ, Turner BJ, Guest WC, Pokrishevsky E, O’Neill MA, Yanai A, Silverman JM, Zeineddine R, Corcoran L, Kumita JR, Luheshi LM, Yousefi M, Coleman BM, Hill AF, Plotkin SS, Mackenzie IR, Cashman NR (2014). Intercellular propagated misfolding of wild-type Cu/Zn superoxide dismutase occurs via exosome-dependent and -independent mechanisms. Proc. Natl. Acad. Sci. USA.

[13] Forsberg K, Jonsson PA, Andersen PM, Bergemalm D, Graffmo KS, Hultdin M, Jacobsson J, Rosquist R, Marklund SL, Brännström T (2010). Novel antibodies reveal inclusions containing non-native SOD1 in sporadic ALS patients. PLoS One.

[14] Medinas DB, Rozas P, MartínezTraub F, Woehlbier U, Brown RH, Bosco DA, Hetz C (2018). Endoplasmic reticulum stress leads to accumulation of wild-type SOD1 aggregates associated with sporadic amyotrophic lateral sclerosis. Proc. Natl. Acad. Sci. USA.

[15] Proctor EA, Fee L, Tao Y, Redler RL, Fay JM, Zhang Y, Lv Z, Mercer IP, Deshmukh M, Lyubchenko YL, Dokholyan NV (2016). Nonnative SOD1 trimer is toxic to motor neurons in a model of amyotrophic lateral sclerosis. Proc. Natl. Acad. Sci. USA.

[16] Zhu C, Beck MV, Griffith JD, Deshmukh M, Dokholyan NV (2018). Large SOD1 aggregates, unlike trimeric SOD1, do not impact cell viability in a model of amyotrophic lateral sclerosis. Proc. Natl. Acad. Sci. USA.

[17] Vaquer-Alicea J, Diamond MI (2019). Propagation of protein aggregation in neurodegenerative diseases. Annu. Rev. Biochem..

[18] Vassall KA (2011). Decreased stability and increased formation of soluble aggregates by immature superoxide dismutase do not account for disease severity in ALS. Proc. Natl. Acad. Sci. USA.

[19] Furukawa Y, Fu R, Deng H-X, Siddique T, O'Halloran TV (2006). Disulfide cross-linked protein represents a significant fraction of ALS-associated Cu, Zn-superoxide dismutase aggregates in spinal cords of model mice. Proc. Natl. Acad. Sci. USA.

[20] Toichi K, Yamanaka K, Furukawa Y (2013). Disulfide scrambling describes the oligomer formation of superoxide dismutase (SOD1) proteins in the familial form of amyotrophic lateral sclerosis. J. Biol. Chem..

[21] Ivanova MI, Sievers SA, Guenther EL, Johnson LM, Winkler DD, Galaleldeen A, Sawaya MR, Hart PJ, Eisenberg DS (2014). Aggregation-triggering segments of SOD1 fibril formation support a common pathway for familial and sporadic ALS. Proc. Natl. Acad. Sci. USA.

[22] Sangwan S, Zhao A, Adams KL, Jayson CK, Sawaya MR, Guenther EL, Pan AC, Ngo J, Moore DM, Soriaga AB, Do TD, Goldschmidt L, Nelson R, Bowers MT, Koehler CM, Shaw DE, Novitch BG, Eisenberg DS (2017). Atomic structure of a toxic, oligomeric segment of SOD1 linked to amyotrophic lateral sclerosis (ALS). Proc. Natl. Acad. Sci. USA.

[23] Sangwan S, Sawaya MR, Murray KA, Hughes MP, Eisenberg DS (2018). Atomic structures of corkscrew-forming segments of SOD1 reveal varied oligomer conformations. Protein Sci..

[24] DiDonato M, Craig L, Huff ME, Thayer MM, Cardoso RMF, Kassmann CJ, Lo TP, Bruns CK, Powers ET, Kelly JW, Getzoff ED, Tainer JA (2003). ALS mutants of human superoxide dismutase form fibrous aggregates via framework destabilization. J. Mol. Biol..

[25] Wiedau-Pazos M, Goto Joy J, Rabizadeh S, Gralla Edith B, Roe James A, Lee Michael K, Valentine Joan S, Bredesen Dale E (1996). Altered reactivity of superoxide dismutase in familial amyotrophic lateral sclerosis. Science.

[26] Yim MB, Kang JH, Yim HS, Kwak HS, Chock PB, Stadtman ER (1996). A gain-of-function of an amyotrophic lateral sclerosis-associated Cu, Zn-superoxide dismutase mutant: An enhancement of free radical formation due to a decrease in Km for hydrogen peroxide. Proc. Natl. Acad. Sci. USA.

[27] Ghadge GD, Lee JP, Bindokas VP, Jordan J, Ma L, Miller RJ, Roos RP (1997). Mutant superoxide dismutase-1-linked familial amyotrophic lateral sclerosis: Molecular mechanisms of neuronal death and protection. J. Neurosci..

[28] Estévez AG, Crow JP, Sampson JB, Reiter C, Zhuang Y, Richardson GJ, Tarpey MM, Barbeito L, Beckman JS (1999). Induction of nitric oxide-dependent apoptosis in motor neurons by zinc-deficient superoxide dismutase. Science.

[29] Liu D, Wen J, Liu J, Li L (1999). The roles of free radicals in amyotrophic lateral sclerosis: Reactive oxygen species and elevated oxidation of protein, DNA, and membrane phospholipids. FASEB J..

[30] Kitamura F, Fujimaki N, Okita W, Hiramatsu H, Takeuchi H (2011). Structural instability and Cu-dependent pro-oxidant activity acquired by the apo form of mutant SOD1 associated with amyotrophic lateral sclerosis. Biochemistry.

[31] Fujimaki N, Nishiya K, Miura T, Nakabayashi T (2016). Acquisition of pro-oxidant activity of fALS-linked SOD1 mutants as revealed using circular dichroism and UV-resonance Raman spectroscopy. Chem. Phys..

[32] Ohyama T, Kuroi K, Wakabayashi T, Fujimaki N, Nakabayashi T (2020). Enhancement of oxidative reaction by the intramolecular electron transfer between the coordinated redox-active metal ions in SOD1. J. Phys. Chem. B.

[33] Fujimaki N, Kitamura F, Takeuchi H (2013). Pro-oxidant copper-binding mode of the apo form of ALS-linked SOD1 mutant H43R denatured at physiological temperature. Biochemistry.

[34] Fujimaki N, Miura T, Nakabayashi T (2016). The structural analysis of the pro-oxidant copper-binding site of denatured apo-H43R SOD1 and the elucidation of the origin of the acquisition of the pro-oxidant activity. Phys. Chem. Chem. Phys..

[35] Takahashi A, Nagao C, Murakami K, Kuroi K, Nakabayashi T (2020). Effects of molecular crowding environment on the acquisition of toxic properties of wild-type SOD1. Biochim. Biophys. Acta Gen. Subj..

[36] Nagao C, Kuroi K, Wakabayashi T, Nakabayashi T (2020). Pro-oxidant activity of an ALS-linked SOD1 mutant in Zn-deficient form. Molecules.

[37] Fisher CL, Cabelli DE, Tainer JA, Hallewell RA, Getzoff ED (1994). The role of arginine 143 in the electrostatics and mechanism of Cu, Zn superoxide dismutase: Computational and experimental evaluation by mutational analysis. Proteins.

[38] Hart PJ, Balbirnie MM, Ogihara NL, Nersissian AM, Weiss MS, Valentine JS, Eisenberg D (1999). A structure-based mechanism for copper−zinc superoxide dismutase. Biochemistry.

[39] Furukawa Y, Anzai I, Akiyama S, Imai M, Cruz FJC, Saio T, Nagasawa K, Nomura T, Ishimori K (2016). Conformational disorder of the most immature Cu, Zn-superoxide dismutase leading to amyotrophic lateral sclerosis. J. Biol. Chem..

[40] Banci L, Felli IC, Kümmerle R (2002). Direct detection of hydrogen bonds in monomeric superoxide dismutase: Biological implications. Biochemistry.

[41] Jagaraj CJ, Parakh S, Atkin JD (2021). Emerging evidence highlighting the importance of redox dysregulation in the pathogenesis of amyotrophic lateral sclerosis (ALS). Front. Cell. Neurosci..

[42] Carrì MT, Ferri A, Cozzolino M, Calabrese L, Rotilio G (2003). Neurodegeneration in amyotrophic lateral sclerosis: the role of oxidative stress and altered homeostasis of metals. Brain Res. Bull..

[43] Poon HF, Hensley K, Thongboonkerd V, Merchant ML, Lynn BC, Pierce WM, Klein JB, Calabrese V, Butterfield DA (2005). Redox proteomics analysis of oxidatively modified proteins in G93A-SOD1 transgenic mice—a model of familial amyotrophic lateral sclerosis. Free Radic. Biol. Med..

[44] Chi L, Ke Y, Luo C, Gozal D, Liu R (2007). Depletion of reduced glutathione enhances motor neuron degeneration in vitro and in vivo. Neuroscience.

[45] Bakavayev S, Chetrit N, Zvagelsky T, Mansour R, Vyazmensky M, Barak Z, Israelson A, Engel S (2019). Cu/Zn-superoxide dismutase and wild-type like fALS SOD1 mutants produce cytotoxic quantities of H2O2 via cysteine-dependent redox short-circuit. Sci. Rep..

[46] Ferri A, Cozzolino M, Crosio C, Nencini M, Casciati A, GrallaEdith B, Rotilio G, Valentine Joan S, CarrìMaria T (2006). Familial ALS-superoxide dismutases associate with mitochondria and shift their redox potentials. Proc. Natl. Acad. Sci. USA.

[47] Marden JJ, Harraz MM, Williams AJ, Nelson K, Luo M, Paulson H, Engelhardt JF (2007). Redox modifier genes in amyotrophic lateral sclerosis in mice. J. Clin. Invest..

[48] Kaneko M, Noguchi T, Ikegami S, Sakurai T, Kakita A, Toyoshima Y, Kambe T, Yamada M, Inden M, Hara H, Oyanagi K, Inuzuka T, Takahashi H, Hozumi I (2015). Zinc transporters ZnT3 and ZnT6 are downregulated in the spinal cords of patients with sporadic amyotrophic lateral sclerosis. J. Neurosci. Res..

[49] Crow JP, Sampson JB, Zhuang Y, Thompson JA, Beckman JS (1997). Decreased zinc affinity of amyotrophic lateral sclerosis-associated superoxide dismutase mutants leads to enhanced catalysis of tyrosine nitration by peroxynitrite. J. Neurochem..

[50] Hayward LJ, Rodriguez JA, Kim JW, Tiwari A, Goto JJ, Cabelli DE, Valentine JS, Brown RH (2002). Decreased metallation and activity in subsets of mutant superoxide dismutases associated with familial amyotrophic lateral sclerosis. J. Biol. Chem..

