# Supplementary material for: SOD1 gains pro-oxidant activity upon aberrant oligomerization: change in enzymatic activity by intramolecular disulfide bond cleavage
Source: Sci Rep. 2022 Jul 11;12:11750. doi: 10.1038/s41598-022-15701-w (PMC9273606; doi:10.1038/s41598-022-15701-w)
Supplement: Supplementary file 1 — Supplementary Information. [file 41598_2022_15701_MOESM1_ESM.docx]

**Supplementary Information for**

SOD1 gains pro-oxidant activity upon aberrant oligomerization: change in enzymatic activity by intramolecular disulfide bond cleavage

Kosuke Yamazaki, Shinya Tahara, Takumi Ohyama, Kunisato Kuroi, Takakazu Nakabayashi

Corresponding authors: Takakazu Nakabayashi, Shinya Tahara

Email: takakazu.nakabayashi.e7@tohoku.ac.jp (TN); shinya.tahara.c6@tohoku.ac.jp (ST)

**This PDF file includes:**

Figures S1 to S7


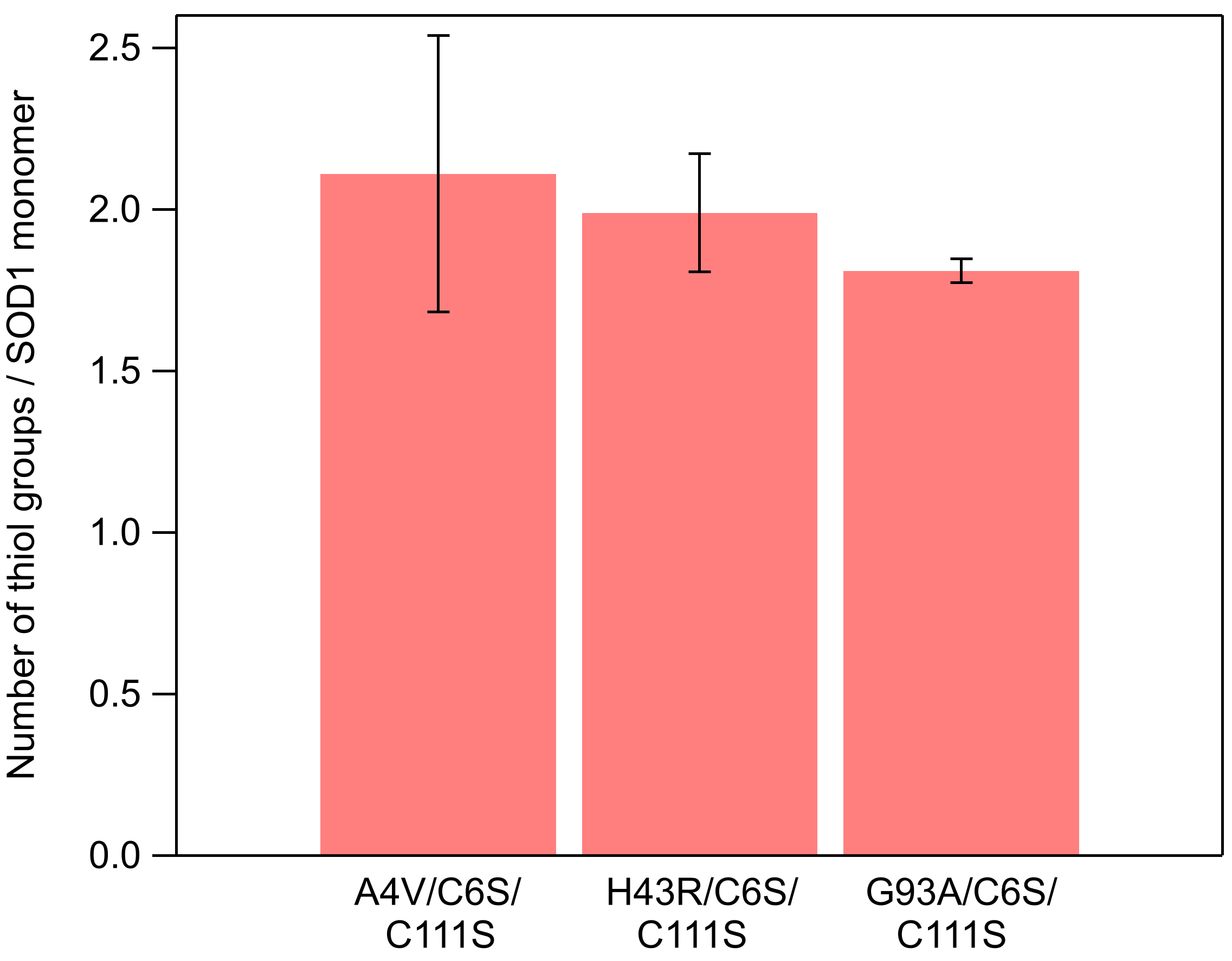


**Figure S1.** The number of thiol groups per monomer unit of apo-A4V/C6S/C111S, apo-H43R/C6S/C111S, and apo-G93A/C6S/C111S after the incubation for 24 h at 45 °C in the presence of dithiothreitol (DTT). Error bars are SE (*n* = 3). The 5,5’-dithio-bis-(2-nitrobenzoic acid) (DTNB) assay was employed to evaluate the number of thiol groups. The number of thiol groups was almost 2 for all the mutants, indicating that the sole intramolecular disulfide bond (Cys57-Cys146) was almost completely reduced after the incubation with DTT. The detailed method is described in the Materials and Methods section.


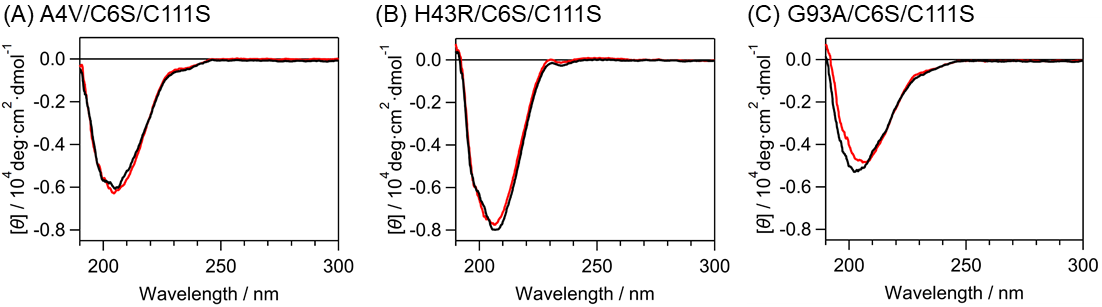


**Figure S2.** CD spectra of (A) apo-A4V/C6S/C111S, (B) apo-H43R/C6S/C111S, and (C) apo-G93A/C6S/C111S before (black) and after (red) the incubation for 90 min at 37 °C. The CD spectra hardly changed after the incubation, indicating that these mutants did not undergo the denaturation under this incubation condition.


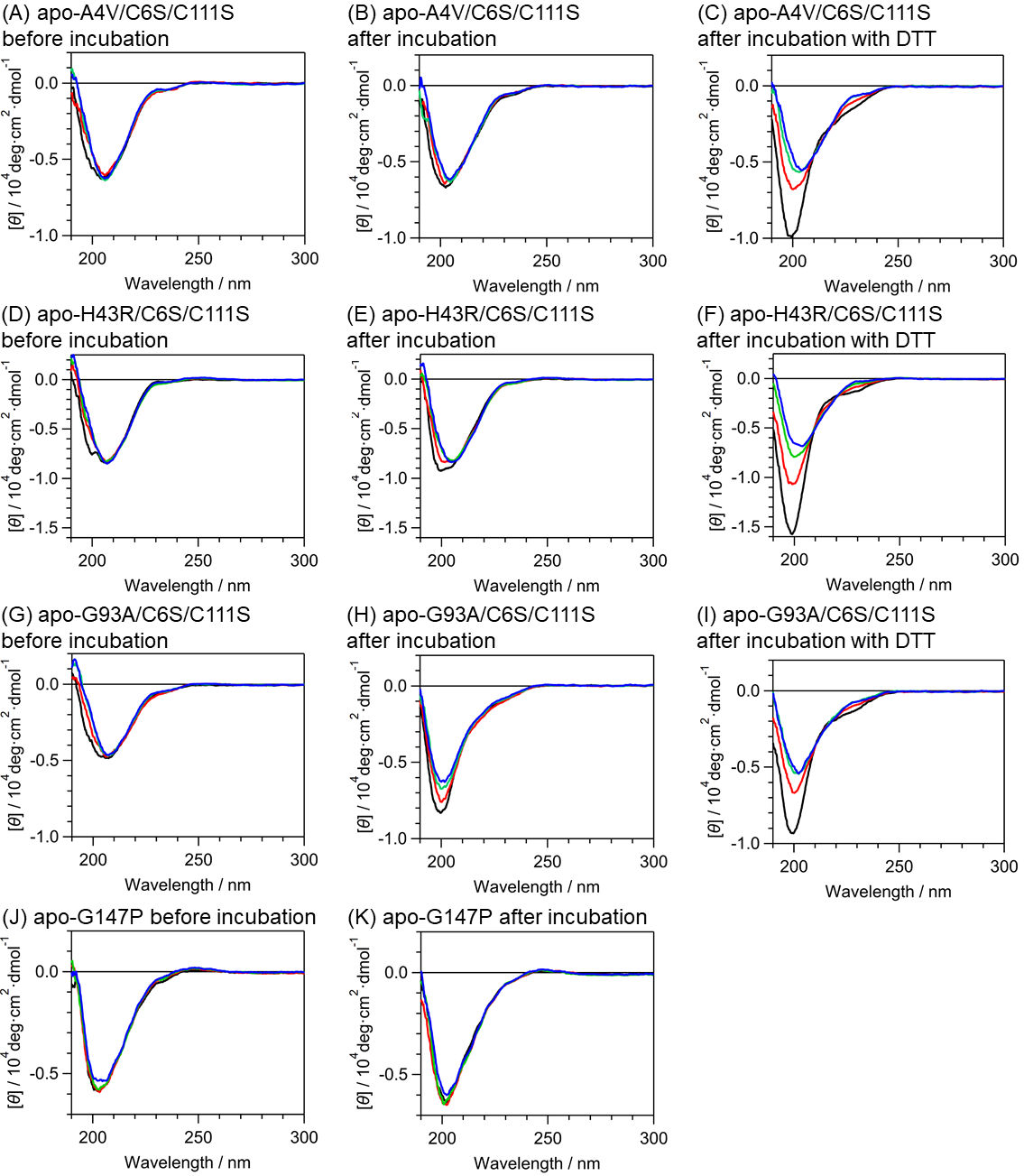


**Figure S3.** CD spectra of the SOD1 mutants before (black) and after the addition of 1- (red), 2- (green), and 3- (blue) fold molar excess of Cu ions over the monomer. The spectra of apo-A4V/C6S/C111S before and after the incubation are shown in Panels (A) and (B), respectively, and that after the incubation with DTT is shown in Panel (C). Similarly, the spectra of apo-H43R/C6S/C111S and apo-G93A/C6S/C111S are shown in Panels (D-I). The spectra of apo-G147P before and after the incubation are shown in Panels (J) and (K), respectively. All the CD spectra of the C6S/C111S mutants (Panels A-I) changed with increasing the Cu ion concentration up to 2-fold molar excess, reflecting structural changes associated with Cu ion binding. The spectra showed only little changes when the Cu ion concentration was increased from 2 to 3-fold molar excess, confirming that the metal-binding sites were almost entirely occupied by Cu ions. The CD spectra of apo-G147P before and after the incubation were similar to those of other mutants after Cu ion binding and showed negligible changes even after adding 3-fold molar excess of Cu ions.


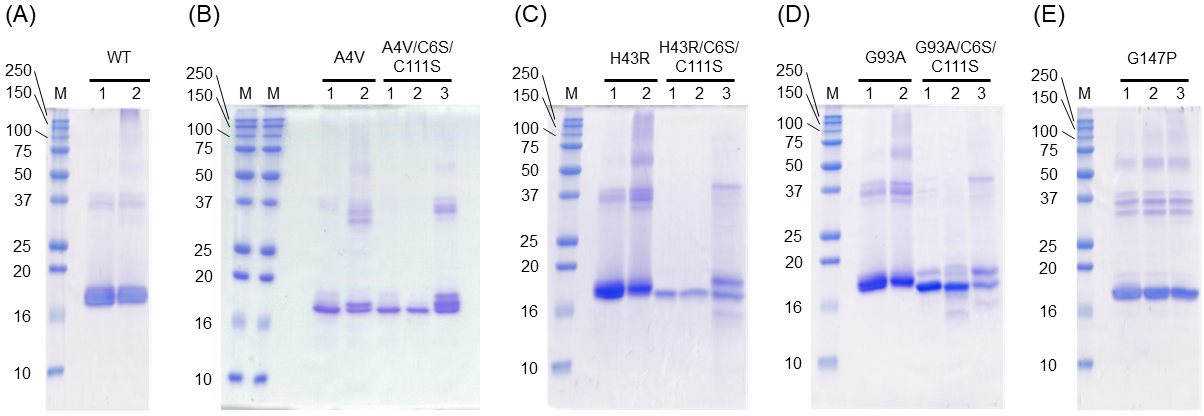


**Figure S4.** Full-length SDS-PAGE gel images of all the mutants with the molecular weight markers. (A) apo-WT, (B) apo-A4V and apo-A4V/C6S/C111S mutants, (C) apo-H43R and apo-H43R/C6S/C111S mutants, (D) apo-G93A and apo-G93A/C6S/C111S mutants, and (E) apo-G147P mutant. In Panels (A-E), the lanes indicated with “M” show the molecular weight makers. In Panels (A-D), lanes: 1, before the incubation; 2, after the incubation; 3, after the incubation with 10 mM DTT. In panel (E), lanes: 1, before the incubation; 2, after the incubation for 90 min; 3, after the incubation for 24 h.

**
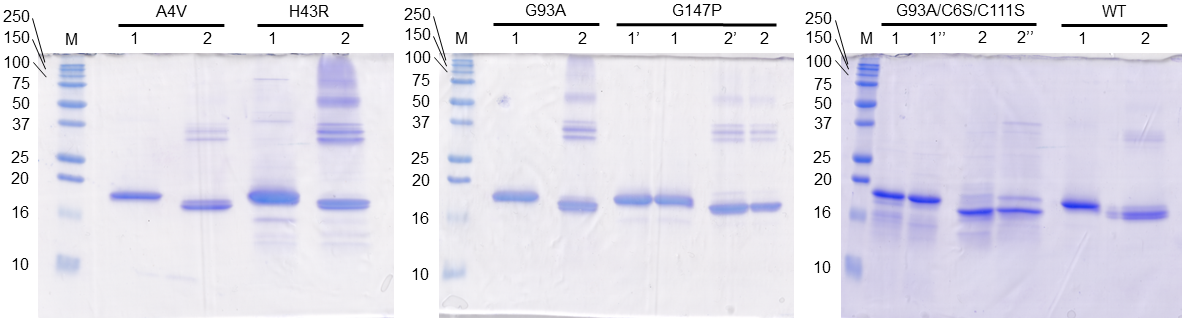
**

**Figure S5.** Comparison of reducing and non-reducing SDS-PAGE results of apo-A4V, apo-H43R, apo-G93A, apo-G147P, apo-G93A/C6S/C111S, and apo-WT SOD1. The lanes indicated with “M” show the molecular weight makers. Lanes: 1, reducing SDS-PAGE of the sample after the incubation; 2, non-reducing SDS-PAGE of the sample after the incubation; 1’, reducing SDS-PAGE of the sample before the incubation; 2’, non-reducing SDS-PAGE of the sample before the incubation; 1’’, reducing SDS-PAGE of the sample after the incubation with 10 mM DTT; 2’’, non-reducing SDS-PAGE of the sample after the incubation with 10 mM DTT. The non-reducing SDS-PAGE of apo-WT, apo-A4V, apo-H43R, apo-G93A, apo-G147P, and DTT-treated apo-G93A/C6S/C111S exhibited bands due to oligomers at around and above 37 kDa, while their reducing SDS-PAGE results showed only a band due to monomers. These results indicate that the oligomers are generated by formations of intermolecular disulfide bonds.


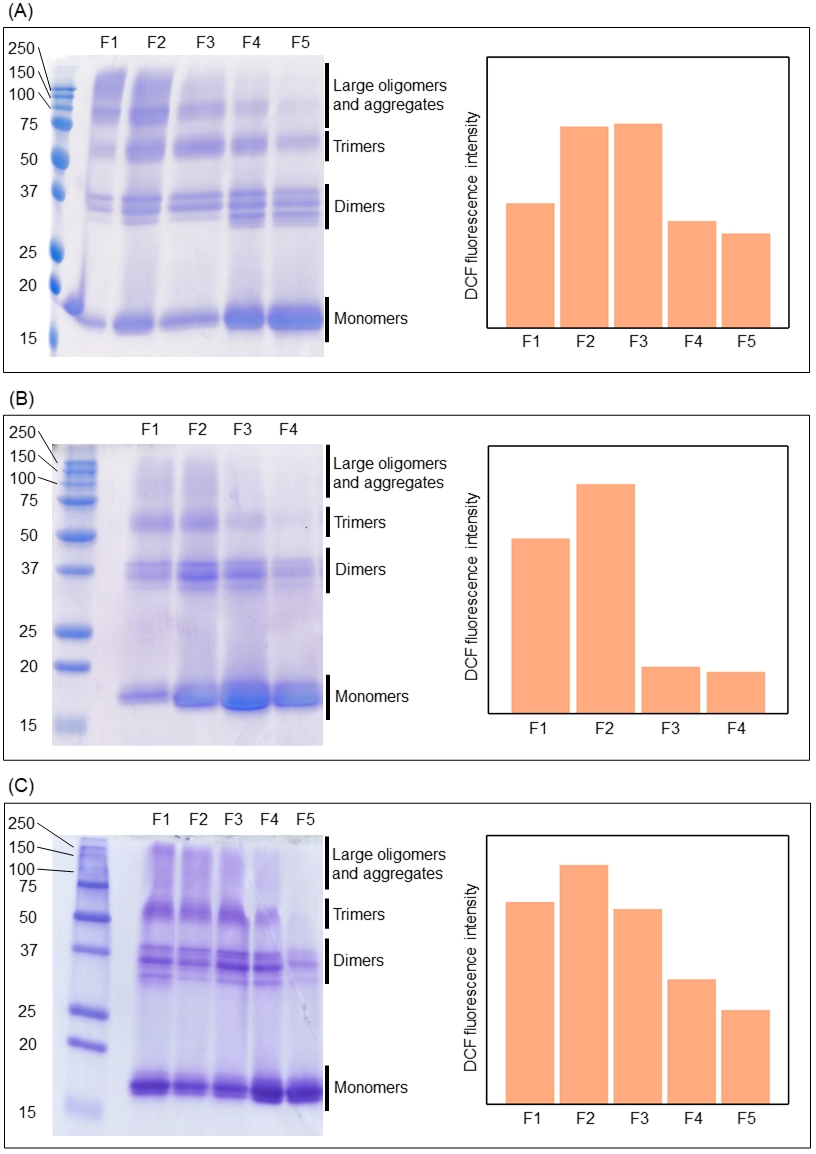


**Figure S6.** Non-reducing SDS-PAGE and DCF fluorescence measurements of the eluted fractions obtained from the size-exclusion chromatography of apo-G93A after the incubation for 90 min at 37 °C. The results of three independent experiments are shown in Panels (A), (B), and (C). The eluted solutions of size-exclusion chromatography were consecutively collected in five (or four) tubes and labeled F1, F2, F3, F4, and F5 (or F1, F2, F3, and F4) according to the order of the elution volume. We note that oligomers with different molecular weights were not completely separated by size-exclusion chromatography and the oligomer composition of the eluted fractions varied in each experiment, as shown in the SDS-PAGE results in Panels (A-C). The SDS-PAGE results in Panel (A) indicate that the concentrations of trimers and dimers in F2 and F3 are higher than those in F1, F4, and F5. The concentrations of large oligomers and aggregates are highest in F1, and monomers are dominant in F4 and F5. The DCF fluorescence intensities of F2 and F3 were stronger than those of F1, F4, and F5, meaning that the trimers and dimers exhibit stronger pro-oxidant activity than aggregates, larger oligomers, and monomers.


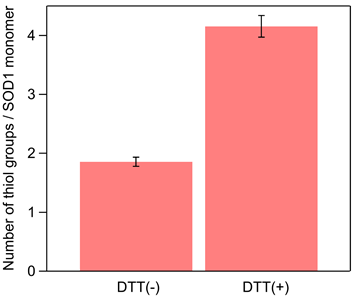


**Figure S7.** The number of thiol groups per apo-G93A monomer before and after the DTT treatment. Error bars are SE (*n* = 3). The number of thiol groups evaluated by the DTNB assay was almost 2 (Cys6 and Cys111) after the incubation for 24 h at 4 °C without DTT, indicating that the disulfide bond (Cys57-Cys146) remained intact. There were about 4 thiol groups after the incubation with DTT, confirming the cleavage of the Cys57-Cys146 bond.
